# Perilipin Expression Reveals Adipogenic Potential of hADSCs inside Superporous Polymeric Cellular Delivery Systems

**DOI:** 10.1155/2014/830791

**Published:** 2014-05-04

**Authors:** Sorina Dinescu, Bianca Galateanu, Adriana Lungu, Eugen Radu, Sorin Nae, Horia Iovu, Marieta Costache

**Affiliations:** ^1^Department of Biochemistry and Molecular Biology, University of Bucharest, 91-95 Splaiul Independentei, 050095 Bucharest, Romania; ^2^Advanced Polymer Materials Group, Department of Bioresources and Polymer Science, University Politehnica of Bucharest, 149 Calea Victoriei, 010072 Bucharest, Romania; ^3^Molecular Biology and Pathology Research Lab “Molimagex”, University Hospital Bucharest, 169 Splaiul Independentei, 050098 Bucharest, Romania; ^4^Faculty of Medicine, Ovidius University of Constanta, 1 Aleea Universitatii, 900527 Constanta, Romania

## Abstract

Recent progress in tissue engineering and regenerative medicine envisages the use of cell-scaffold bioconstructs to best mimic the natural *in vivo* microenvironment. Our aim was not only to develop novel 3D porous scaffolds for regenerative applications by the association of gelatin (G), alginate (A), and polyacrylamide (PAA) major assets but also to evaluate their *in vitro* potential to support human adipose-derived stem cells (hADSCs) adipogenesis. G-A-PAA biomatrix investigated in this work is an interesting substrate combining the advantages of the three individual constituents, namely, biodegradability of G, hydrophilicity of A and PAA, superior elasticity at compression with respect to the G-A and PAA controls, and the capacity to generate porous scaffolds. hADSCs inside these novel interpenetrating polymer networks (IPNs) were able to populate the entire scaffold structure and to display their characteristic spindle-*like* shape as a consequence of a good interaction with G component of the matrices. Additionally, hADSCs proved to display the capacity to differentiate towards mature adipocytes, to accumulate lipids inside their cytoplasm, and to express perilipin late adipogenic marker inside novel IPNs described in this study. On long term, this newly designed biomatrix aims to represent a stem cell delivery system product dedicated for modern regenerative strategies.

## 1. Introduction

Modern tissue engineering (TE) applications require the correlation between the composition, structure, and characteristics of the material and the biological component. The interaction of the scaffold with cells, fluids, and tissues is strongly dependent on the chemistry of the material, since the physicochemical features of the material can decisively influence cell adherence.

Polymers are versatile natural and synthetic compounds displaying a large panel of properties that make them suitable for a wide range of TE applications. Despite their specific biodegradability and biocompatibility, some materials do not possess appropriate mechanical properties or biodegradation rate. In this context, we recently developed and investigated various multicomponent scaffolds based on semi- and interpenetrating polymer networks (IPNs) by combining natural, synthetic, biodegradable, and/or nonbiodegradable macromolecular components such as gelatin-alginate [1, 2], gelatin-alginate-polyacrylamide (PAA) [3], fibroin-PAA [4], gelatin-poly(2-hydroxyethyl methacrylate) (PHEMA) [5], and collagen-sericin [6, 7]. The underlying principle was that the natural polymers (i.e., collagen, gelatin, and alginate) would impair biodegradability to the resulting bi- or tricomponent scaffolds, while displaying improved overall properties. Furthermore, the presence of collagen or gelatin in a scaffold's formulation confers cell adhesion properties, while also ensuring enzymatic biodegradation. In addition, macromolecular elements with high water affinity, such as PAA and alginate, enhance the degradation rate of multicomponent scaffolds due to improved accessibility of the substrate to hydrolytic attack [3, 5], thus improving the overall water affinity of such multicomponent scaffolds. Taking together all these features, we recently synthesized and characterized a tricomponent gelatin-alginate-PAA system as appealing substrates for soft tissue regeneration [3].

In a dynamic view, adipose tissue (AT) through its cellular component, the adipocytes, generates a wide range of signal molecules such as growth factors, proteins related to the immune system, and adipokines [8]. Particularly, subcutaneous adipose depots are accessible and abundant, in contrast with the bone marrow (BM), the traditional mesenchymal stem cells (MSCs) harvesting source. In this perspective, AT has become an attractive option for adipose-derived stem cells isolation (ADSCs). ADSCs found in the stromal-vascular fraction (SVF) of the AT have the ability to differentiate into cells of several lineages such as adipocytes, osteoblasts, chondrocytes, myocytes, endothelial cells, hematopoietic cells, hepatocytes, and neuronal cells [9–18].

The main promoters of adipogenic differentiation, PPAR*γ* and C/EBP*α*, act synergistically to activate transcription of genes that produce the adipocyte phenotype, although hormones are required for terminal differentiation [19, 20]. Mature adipocytes synthesize AT-specific products, such as adipocyte fatty acid-binding protein (aP2) and perilipin [21]. Lipid droplet-associated protein, perilipin, coats lipid droplets in mature adipocytes and acts as a protective layer against the physiological lipases.

In this context, our aim was not only to develop a combinatory approach of the gelatin, alginate, and PAA major assets to design novel 3D porous scaffolds for soft tissue regenerative applications, but also to evaluate their* in vitro* potential to support hADSCs differentiation towards mature and functional adipocytes. On long term, this newly designed biomatrix aims to represent a stem cell delivery system product dedicated for modern regenerative strategies. Therefore, essential functional properties such as the water affinity, the mechanical properties, and the enzymatic degradation of the porous tricomponent gelatin-alginate-PAA scaffolds were evaluated. In addition, cell behavior and distribution, as well as the potential to accumulate lipid droplets and to express late adipogenic markers such as perilipin during* in vitro* adipogenesis, were also assessed.

## 2. Materials and Methods

### 2.1. Materials

Gelatin B (further named Gel) from bovine skin (Sigma) was used as 20% (w/v) aqueous solution. Sodium alginate (SA) was used as 4% (w/v) aqueous solution. Acrylamide (AAm) for electrophoresis >99% (HPLC), N,N′-methylenebis(acrylamide) (MBA) 99%, triethanolamine (TEA), ammonium persulfate (APS), glutaric aldehyde (GA) as aqueous solution 25%, and calcium chloride anhydrous (CaCl_2_) were purchased from Sigma and used without further purification. Ethylene diamine tetra-acetic acid (tetrasodium salt tetrahydrate) (EDTA) from Sigma-Aldrich was used as received. Sodium azide (99%) was purchased from Avocado Research Chemicals Ltd. Collagenase type I of* Clostridium histolyticum* with a collagen activity ≥125 units per mg (collagen digestion units) was from Sigma. All the salts necessary to prepare phosphate buffer saline (PBS) were supplied by Sigma-Aldrich.

Human subcutaneous adipose tissue which served as stem cells source for this study was harvested from adult patients undergoing elective abdominoplasty. All the subjects offered their written informed consent to participate in this study and none of them had diabetes or severe systemic illness or was taking medication known to affect adipose tissue metabolism. All the medical procedures were performed in compliance with the Helsinki Declaration, with the approval of the Emergency Hospital for Plastic Surgery and Burns Ethical Committee (Reference number 3076/10.06.2010). hADSCs were manipulated using sterile Thermo Scientific Nunc labware disposables. MesenPRO RS culture medium and StemPro Adipogenesis Differentiation Kit (Gibco, Life Technologies, Foster City, CA) were used to propagate and differentiate hADSCs. Glutaraldehyde, bovine serum albumin (BSA), Triton X-100, and Oil Red O dye were purchased from Sigma-Aldrich Co. RNA extraction was performed using TRIzol Reagent provided by Invitrogen, Foster City, CA, USA, and the qPCR LightCycler FastStart DNA Master SYBR Green I Kit was provided by Roche, Mannheim, Germany. All the primary and secondary antibodies used in this study were purchased from Santa Cruz Biotechnology, Inc. (Heidelberg, Germany).

### 2.2. Methods

#### 2.2.1. Scaffold Synthesis

IPNs based on G, SA, and PAA were prepared using a previously described three-step procedure [3] which was adapted for this particular study. Briefly, semi-IPNs were initially generated by the free-radical copolymerization of AA and MBA in water in the presence of G and SA. A weight ratio of 14 : 1 : 20 between G, SA, and AA was used, with a total solid content (*T*%) of 21%. A redox initiating system based on APS (1% molar with respect to AA and MBA) and TEA (1/2 molar with respect to APS) was used to perform the polymerization reaction at room temperature (RT). The molar ratio between MBA and AA was 1.8 : 100. A copolymerization stock solution was prepared through the dissolution of AA, MBA, and the corresponding amount of APS in distilled water, under stirring, at RT. 1 mL of this solution was further mixed with 8 mL of G solution and with 1 mL of SA solution, at 40°C. The resulting mixture was degassed using an ultrasound bath (Elma S 30 H, Elmasonic) for 15 minutes at 40°C and finally TEA was added under vigorous stirring. The copolymerization reaction of AA and MBA was allowed, for 24 hours, at RT and, consequently, semi-IPNs consisting in crosslinked PAA and uncrosslinked G and SA were obtained. Furthermore, the materials were cooled for 2 hours at 4°C, to allow physical gelation of G. In a second step, the crosslinking of G was performed through their immersion in GA 0.5% for 24 hours, at RT. The third phase of the synthesis consisted in the crosslinking of SA by immersing the samples for 24 hours in a 1% CaCl_2_ aqueous solution. As a result, calcium alginate (A) was formed. The G-A-PAA hydrogel was further extracted in distilled water at 40°C for four days. Gravimetric measurement was used to confirm the success of the IPNs formation. The synthesis of porous scaffolds was performed through a freeze-drying treatment as previously reported [6].

In order to investigate the potential advantages of the tricomponent IPN, control G-A and PAA hydrogels with the same *T*% (21%) were synthesized following similar procedures and submitted to lyophilization to generate porous materials. While PAA was obtained through the redox initiated free-radical polymerization of the corresponding monomer and crosslinker (molar ratio MBA/AA of 1.8/100, *T* = 21%), G-A hydrogels were obtained using a three-step crosslinking. Briefly, the preparation of the bicomponent G-SA solution (Gel/SA of 14/1 and *T* = 21%) was followed by the physical gelation of G (2 hours at 4°C). Then, the crosslinking of G was performed through the immersion of the specimens in GA 0.5% for 24 hours, at RT. The third phase consisted in the crosslinking of SA by immersion of the samples for 24 hours in a 1% CaCl_2_ aqueous solution to generate A. All the control samples were purified as described for G-A-PAA, followed by freeze-drying.

#### 2.2.2. Determination of Water Affinity

The swelling behavior of the G-A-PAA and control freeze-dried hydrogels was investigated in ddw, at 37°C, using the conventional gravimetric method. The swelling ratio (SR) was calculated at predefined time intervals, using the following equation:

(1) $$\begin{array}{c} \text{SR}=\frac{w_{t}-w_{0}}{w_{0}}*100, \end{array}$$

where *w*_*t*_ is the weight of swollen hydrogel at time *t* and *w*_0_ is the initial weight of the dry hydrogel before incubation in ddw. The samples were weighed after the excess of water was removed with filter paper. The maximum swelling degree (MSD) represents the maximum value obtained after reaching equilibrium. After the swelling experiment, the samples were dried and the dry mass was measured in order to allow comparison with the initial dry mass.

#### 2.2.3. Mechanical Properties

The mechanical behaviour of the investigated hydrogels swollen at equilibrium (in ddw at 40°C) was studied using Brookfield CT3 texture analyzer at room temperature. Cylinder samples with the diameter of 10 mm and a thickness of 5 mm were fixed on a base plate and uniaxially compressed by the upper plate connected to a 4500-gram cell. The test speed was set at 0.5 mm/s. A stress versus strain graph was plotted. The compression modulus was calculated from the slope of the linear part of the compression curve at 10% strain.

#### 2.2.4. *In Vitro* Degradation by Collagenase

*In vitro* degradation of the hydrogel G-A-PAA and of the control G-A hydrogel was investigated by incubation of cylindrical freeze-dried samples (Φ = 8 × 5 mm) in collagenase solution, following a procedure reported elsewhere [3, 5]. Briefly, the samples were initially immersed in 0.5 mL Tris-HCl buffer (0.1 M, pH 7.4) in the presence of NaN_3_ (0.0005% w/v) and CaCl_2_ (5 mM) at 37°C. After one hour, 0.5 mL collagenase solution (200 U/mL), dissolved in Tris-HCl buffer, was added. The degradation of G was stopped at predefined time intervals by adding 0.1 mL EDTA solution (0.25 M) and subsequent cooling on ice. Then, the samples were washed three times for 10 minutes with ice-cooled Tris-HCl buffer and three times with double-distilled water. The remaining materials were dried for the determination of the gel fraction (the insoluble polymer fraction remaining after degradation)

(2) $$\begin{array}{c} {\text{Degradation}\cdot \text{extent}}_{\text{ddw}},\%=\frac{w_{0}-w_{f}}{w_{0}}\times 100, \end{array}$$

where *w*_0_ is the initial mass of the sample, while *w*_*f*_ is the weight of the sample after degradation treatment.

#### 2.2.5. *In Vitro* Cell Culture Model and Cell-Scaffold Biohybrid Achievement

hADSCs were isolated as previously described [22] and seeded on top of G-A and G-A-PAA biomatrices at an initial density of 6 × 10^5^ cells/cm^2^ after propagation in MesenPRO RS Medium. In our experiments, the porous 3D constructs resulted after hADSCs populated G-A and G-A-PAA biomatrices were defined as biohybrids. Consequently, they are further addressed as hADSCs/G-A and hADSCs/G-A-PAA, respectively.

Regarding the adipogenic differentiation protocol, the bioconstructs were exposed to proadipogenic conditions for 28 days using StemPro Adipogenesis Differentiation Kit (Gibco, Life Technologies, Foster City, CA). Bioconstructs induction towards the adipogenic lineage was started only after 48 hours after cell seeding into scaffolds. hADSCs potential of differentiation towards the adipogenic lineage was assessed at 3, 7, 14, 21, and 28 days after induction. The time point when the systems were first exposed to the chondrogenic cocktail was considered T0.

#### 2.2.6. Scanning Electron Microscopy Evaluation of Biohybrids during Adipogenesis

The resulting biohybrids were subjected to SEM analysis at 2 days after hADSCs seeding and at 3, 7, 14, 21, and 28 days after adipogenic induction. All the samples were fixed with 2.5% glutaraldehyde (Sigma-Aldrich Co.) for 24 hours at 4°C and then subjected to freeze-drying. The cross-sections were gold-coated and then analyzed using a Quanta Inspect F SEM device equipped with a field emission gun (FEG) with 1.2 nm resolution and with an X-ray energy dispersive spectrometer (EDS).

#### 2.2.7. Intracellular Lipid Accumulation inside Biohybrids Subjected to Adipogenesis

The presence of neutral lipid droplets in the cytoplasm of the differentiating cells inside G-A-PAA and G-A scaffolds was investigated by Oil Red O staining at 3, 7, 14, 21, and 28 days of induction. For this purpose, the biohybrids were fixed overnight in 4% paraformaldehyde. After permeabilization in 2% bovine serum albumin (BSA)/0.1% Triton X-100 solution for 2 hours, both hADSCs/G-A-PAA and hADSCs/G-A bioconstructs were exposed to Oil Red O staining solution for 24 hours at 4°C. The resulting bioconstructs were analyzed by bright-field microscopy using an Olympus IX71 inverted microscope and images were captured with Cell F Imaging Software (Olympus, Hamburg, Germany, 2008).

#### 2.2.8. qPCR Evaluation of Perilipin Late Adipogenic Marker Expression

After hADSCs/G-A and hADSCs/G-A-PAA bioconstructs fragments were exposed to TRIzol Reagent in accordance with the manufacturer's instructions, total RNA was isolated and assessed for concentration, purity (NanoDrop spectrophotometer, Shimadzu, Duisburg, Germany), and integrity on the BioAnalyzer 2100 (Agilent Technologies, Waldbronn, Germany). Late adipogenic marker perilipin expression was evaluated by qPCR, performed on a LightCycler 2.0 carrousel-based system using LightCycler FastStart DNA Master SYBR Green I Kit. The genes coding for ribosomal protein L13A (RPL13A) and TATAA-box binding protein (TBP) were used as reference genes in order to normalize the levels of perilipin adipogenic marker during data processing. Primer sequences used for gene expression assessment are presented in Table 1.

#### 2.2.9. Confocal Fluorescence Microscopy Assessment of Perilipin Protein Expression in 3D Culture Conditions

Perilipin protein expression was assessed at 3, 7, 14, 21, and 28 days after adipogenic induction using a Carl Zeiss LSM710 confocal microscope. Briefly, both hADSCs/G-A and hADSCs/G-A-PAA biohybrids were fixed with 4% PFA, permeabilized with 2% BSA/0.1% Triton X-100 solution, incubated overnight at 4°C with rabbit polyclonal anti-perilipin antibody solution, and finally exposed to TRITC conjugated goat anti-rabbit secondary antibody. Cell nuclei were stained using DAPI dye for 30 min and the resulting labeled constructs were visualized in confocal fluorescence microscopy using a confocal aperture that corresponded to a back-projected size of 1 airy unit. The 405 and 543 nm laser lines were used for excitation and fluorescence emission was detected at 490–515 nm for DAPI and 600–680 nm for TRITC. Carl Zeiss Zen 2010 software version 6.0 was used for image acquisition and analysis.

#### 2.2.10. Statistical Analysis

The spectrophotometric and gene expression data were statistically analyzed using GraphPad Prism 3.03 Software, one-way ANOVA, and Bonferroni test. The experiments were performed with *n* = 3 biological replicates and each data set is presented as the average of three replicates (mean ± standard deviation).

## 3. Results and Discussions

### 3.1. Scaffold Synthesis and Physicochemical Characterization

#### 3.1.1. Swelling Behavior

**Figure 1:**
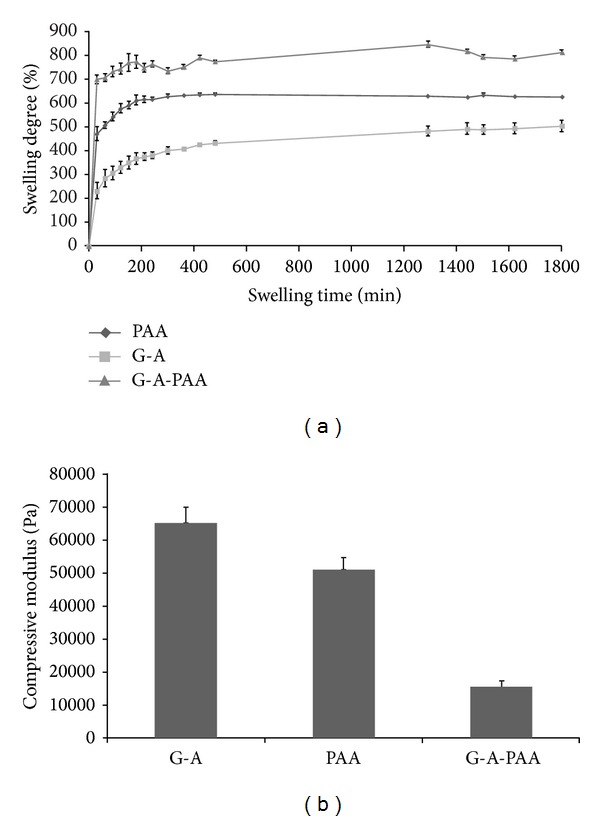
(a) Evolution of the swelling degree of the studied hydrogel and of the control samples, in distilled water, as obtained after 1800 minutes. (b) Effect of the composition on the compressive modulus obtained at room temperature and compressive strain of 10%.

The affinity against water is extremely important since it directly impacts various key properties such as (1) the mechanical behavior, (2) the capacity of the hydrogels to generate porous scaffolds through freeze-drying, and (3) the ability to be loaded with or to deliver water-soluble species. The results of the swelling test are graphically depicted in Figure 1(a). The tricomponent hydrogel G-A-PAA had the highest MSD value, namely, 790% ± 23, when compared to the control hydrogels. The lowest swelling, with a MSD value of 464% ± 6, was noticed for G-A, while the control PAA had a MSD value of 632% ± 4. The swelling is directly determined by the crosslinking density of each network. Interestingly, the combination of the three polymers in a complex IPN with the same *T*% as the control hydrogels increased the affinity for water. This is normal since each additional component, intercalated at molecular level, plays the role of a diluent for the resulting tricomponent IPN. Accordingly, the maximum swelling degree increases. Furthermore, with respect to the time needed to reach the swelling equilibrium, the tricomponent IPN is again the leader, swelling faster than the control materials (PAA needs 300 minutes to reach equilibrium and G-A needs 180 minutes, while G-A-PAA swells in only 90 minutes maximum). In order to estimate if uncrosslinked products are released from the hydrogels during swelling, the final dry mass after the maximum swelling was compared with the initial dry mass of each sample. No significant differences were noticed, confirming the insolubility of the analyzed materials.

#### 3.1.2. Mechanical Behavior

The compression moduli were calculated using the linear part of the stress-strain curves obtained at 10% strain.  Figure 1(b) is representative in this respect and it shows that the compressive moduli decrease in the order G-A > PAA > G-A-PAA. The maximum value (65 ± 5 kPa) was obtained for the IPN G-A. The following value, 52 ± 4 kPa, was obtained for the synthetic hydrogel PAA. The lowest value, 15 ± 2 kPa, was obtained for G-A-PAA. First, these values indicate that the tricomponent IPN is the most elastic hydrogel when compared to the two control materials prepared using the same *T*%. Furthermore, the elasticity perfectly correlates the swelling behavior of the hydrogels increasing with the amount of water from the hydrogel swollen at the maximum: G-A < PAA < G-A-PAA.

#### 3.1.3. Enzymatic Degradation

**Figure 2:**
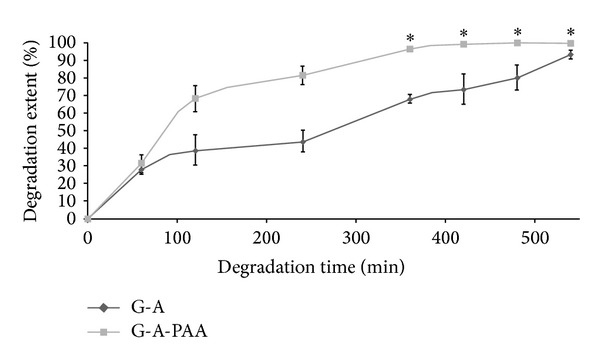
*In vitro* degradation with collagenase I, at 37°C: degradation extents versus degradation time. *These degradation extent values do not indicate a total enzymatic degradation but the disintegration of the scaffolds which become too fragile due to the degradation of G.

Enzymatic degradation was estimated through incubation of the samples in a solution of collagenase. The results are graphically presented in Figure 2. The enzyme degrades the G macromolecules from the IPN. The maximum degradation extent is reached in approximately 6 hours and it corresponds to the degradation of the whole amount of gelatin to water-soluble fractions. Since gelatin is the main component of the studied G-A-PAA IPN (67% G), it appears that the degradation extent should have a value of approximately 67%. However, the tricomponent hydrogel is strongly degraded following the enzymatic attack and it completely loses its integrity after 360 minutes. On the other hand, it can be expected that the presence of the synthetic polymer in the complex IPN increases the stability of the G-A-PAA hydrogel to enzymatic degradation. The obtained results do not contradict the theoretical expectation but indicate that after the degradation of the protein, the remaining network is very fragile and has an increased tendency to disintegrate when immersed in a liquid medium. Furthermore, it can be observed that G-A-PAA is faster degraded when compared to the control G-A (needing 10 hours to reach the maximum degradation level of approximately 94%). Such behavior has a dual explanation: (1) easier access of the enzyme in G-A-PAA due to the larger pores and thinner separation walls and (2) “diluent” effect played by PAA network with respect to the density of G network in the tricomponent IPN. In this context, it can be speculated that if implanted, such a material would degrade in time and only a fragile network of A and PAA will remain embedded in the newly formed tissue.

### 3.2. Cell-Scaffold Interaction and the Adipogenic Potential of hADSCs inside G-A-PAA and G-A IPNs Bioconstructs Behavior during Adipogenesis

#### 3.2.1. hADSCs Morphology and Distribution inside G-A-PAA and G-A Scaffolds during Induced Adipogenic Differentiation

**Figure 3:**
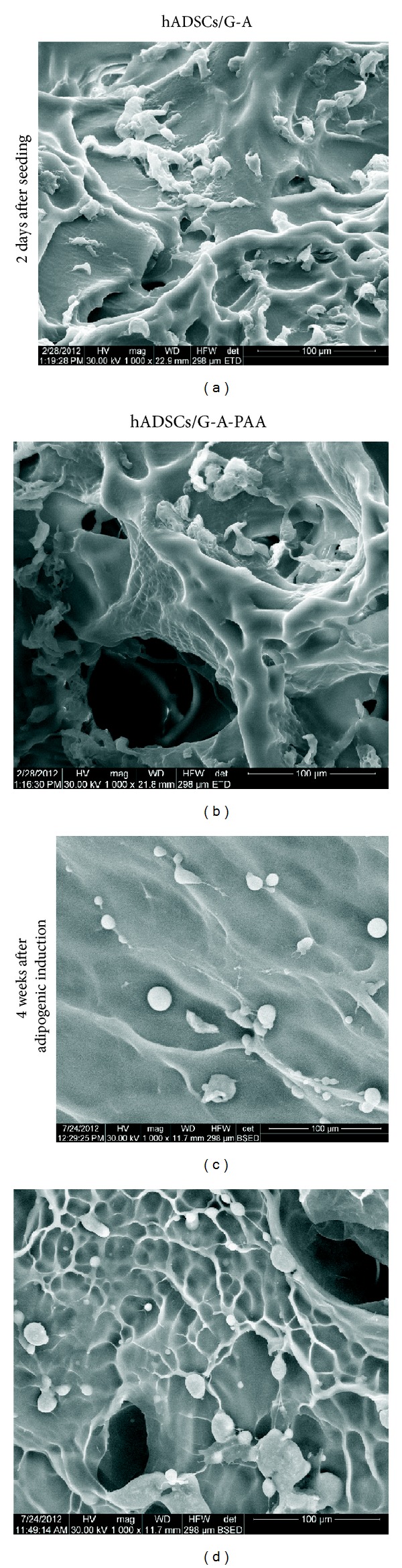
SEM micrographs showing hADSCs morphology and distribution inside hADSCs/G-A-PAA (b, d) and hADSCs/G-A (a, c) bioconstructs at 2 days after cell seeding (a, b) and after 4 weeks of induced* in vitro* adipogenesis (c, d).

SEM analysis of the cross-sections performed in hADSCs/G-A and hADSCs/G-A-PAA biohybrids at 2 days after seeding revealed that both biomatrices were entirely populated with cells, proving the pores interconnectivity pattern of G-A and G-A-PAA scaffolds (Figures 3(a) and 3(b)). In addition, the embedded cells displayed a characteristic spindle*-like* shape inside the tested scaffolds, probably due to their good interaction with the biomaterial (Figures 3(a) and 3(b)).

During 4 weeks of adipogenic induction, hADSCs displayed dramatic changes with respect to their volume and morphology features, most likely as a consequence of the differentiation process that they were undergoing. In Figures 3(c) and 3(d), we presented micrographs of hADSCs/G-A and hADSCs/G-A-PAA biohybrids captured at 28 days after adipogenic induction revealing the adipocyte*-like* spherical shape of the committed hADSCs inside the scaffolds.

#### 3.2.2. Intracellular Lipid Droplet Accumulation inside hADSCs/G-A-PAA and hADSCs/G-A Bioconstructs during Adipogenesis

**Figure 4:**
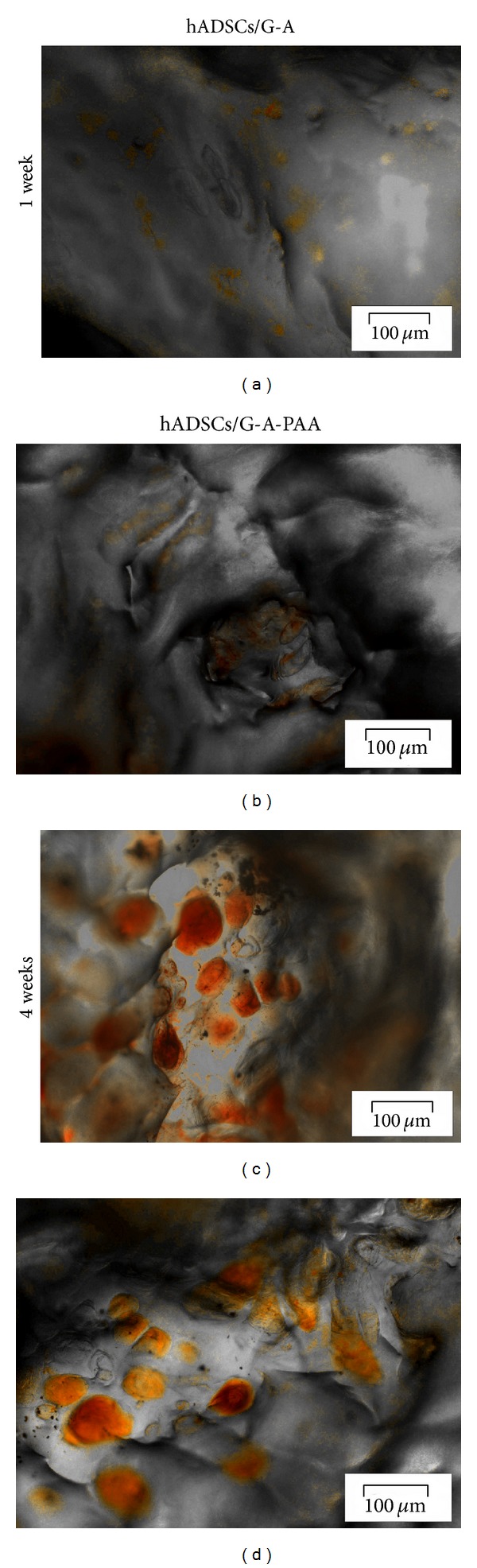
Lipid droplet accumulation inside hADSCs/G-A-PAA (b, d) and hADSCs/G-A (a, c) bioconstructs after 1 week (a, b) and 4 weeks of adipogenic differentiation (c, d), as revealed by Oil Red O staining.

Oil Red O staining of hADSCs/G-A and hADSCs/G-A-PAA biohybrids revealed that after 7 days of adipogenic induction, hADSCs started to accumulate lipids (Figures 4(a) and 4(b)). The small initial lipid containing vesicles started to grow during the exposure of both biohybrids to proadipogenic condition and resulted in large droplets, stained in red with Oil Red O dye (Figures 4(c) and 4(d)), at 4 weeks of experiment.

#### 3.2.3. Perilipin Late Adipogenic Marker Expression Assessment inside hADSCs/G-A-PAA and hADSCs/G-A Bioconstructs during Adipogenesis

**Figure 5:**
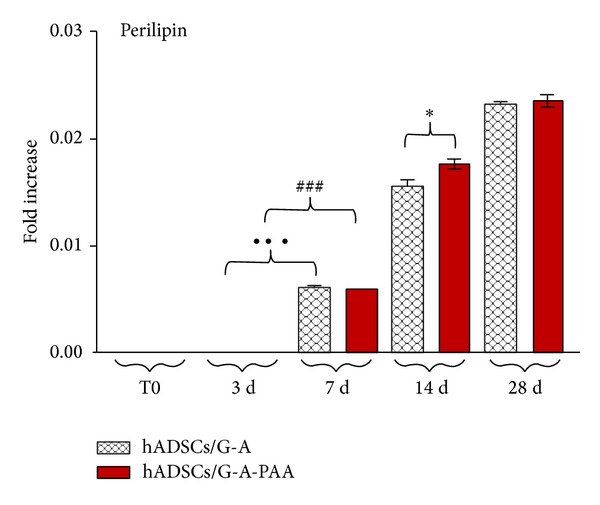
Perilipin gene expression pattern in hADSCs/G-A and hADSCs/G-A-PAA bioconstructs during 28 days of induced adipogenic differentiation, as revealed by qPCR. Statistical meaning (*P < 0.05 (hADSCs/G-A-PAA versus hADSCs/G-A bioconstruct: 14 days); ^###^ *P* < 0.001 (hADSCs/G-A-PAA: 7 days versus 3 days); ^•••^ *P* < 0.001 (hADSCs/G-A: 7 days versus 3 days)).

*Perilipin Gene Expression*. Once the adipogenic signaling is activated via PPAR inducer, the transcription of downstream targets involved in inducing and maintaining the adipocyte phenotype is initiated. Late adipogenic marker perilipin, responsible for lipid storage, was evaluated in terms of gene expression pattern in the conditions of hADSCs/G-A and hADSCs/G-A-PAA 3D cultures during 28 days of induced adipogenic differentiation (Figure 5).

In our conditions, perilipin was first statistically detected (*P* < 0.001) at 7 days after adipogenic induction in both hADSCs/G-A and hADSCs/G-A-PAA biohybrids, as compared to 3 days of adipogenesis. Perilipin levels of gene expression highly increased (*P* < 0.001) between 7 and 14 days of induced adipogenesis in both constructs, suggesting a continuous differentiation process supported by the G-A and G-A-PAA materials and the gradual accumulation of lipids inside differentiating cells. Interestingly, a statistically significant difference (*P* < 0.05) was found between perilipin transcript levels in hADSCs/G-A and hADSCs/G-A-PAA bioconstructs, whereas this difference was not detected at 7 days. Four weeks after adipogenic induction, perilipin mRNA levels significantly increased (*P* < 0.001) in both systems as compared to 14 days, with no statistic difference between hADSCs/G-A-PAA and control biohybrids. According to our results, the increasing profile of perilipin gene expression suggests that an adipogenic differentiation process was active inside the 3D bioconstructs, while similar patterns of gene expression between the hADSCs/G-A and hADSCs/G-A-PAA suggest the nontoxic effect of PAA in the composition of the tested scaffolds.

**Figure 6:**
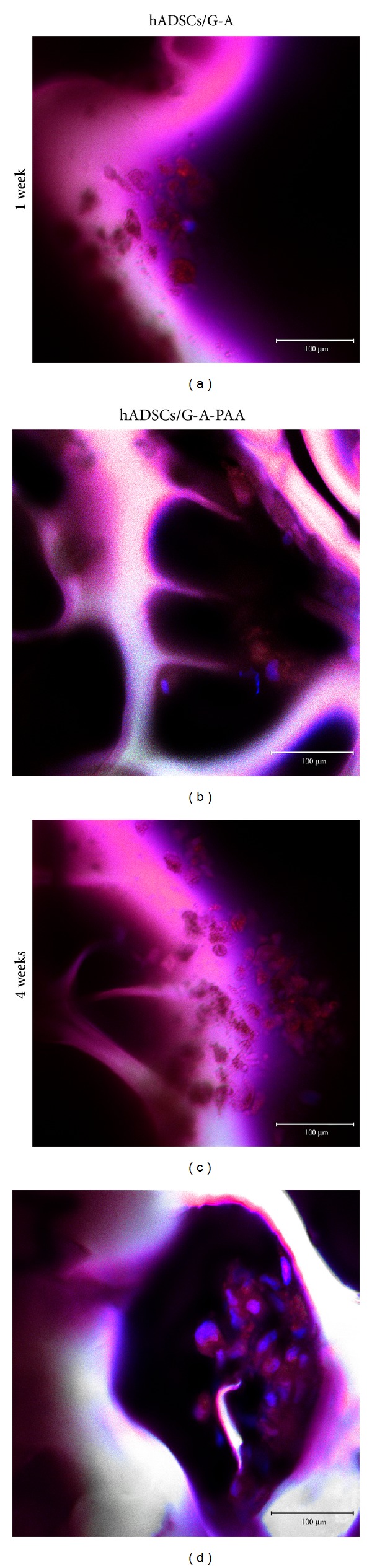
Confocal microscopy micrographs displaying perilipin protein expression in hADSCs/G-A and hADSCs/G-A-PAA biohybrids during adipogenesis.

*Perilipin Protein Expression*. Late adipogenic marker perilipin protein expression was evaluated by confocal fluorescence microscopy at 2 days after seeding and at 3, 7, 14, 21, and 28 days after adipogenic induction of hADSCs/G-A and hADSCs/G-A-PAA biohybrids. Images displaying the earliest positive expression of perilipin at 7 days after induction and images of 4-week differentiating hADSCs were captured at ~90 *μ*m in the scaffolds' depth and are presented in Figure 6.

After one week of adipogenic commitment, TRITC labeled perilipin molecules were observed attached to small vesicles surrounding the blue DAPI stained nucleus in few cells (Figures 6(a) and 6(b)) confirming the Oil Red O staining observations. In addition, 28 days after adipogenic induction, hADSCs inside G-A-PAA's pores highly expressed perilipin as all the nuclei were proved to be surrounded by bright red vesicles (Figures 6(c) and 6(d)). Perilipin expression inside hADSCs/G-A biohybrid was found to be similar.

Our experiments show that, as a result of the adipogenic differentiation progress, adipocytes inside G-A and G-A-PAA biomatrices start to gradually accumulate lipids. Consequently, this increase of intracellular lipids determines the overexpression of perilipin, since perilipin functions as a lipid droplet gatekeeper that controls lipases access to lipids, in a phosphorylation-dependent manner.

## 4. Conclusions

The tricomponent material G-A-PAA investigated in this work is an interesting substrate combining the advantages of the three individual constituents, namely, biodegradability of G, hydrophilicity of A and PAA, superior elasticity at compression with respect to the G-A and PAA controls, and, nevertheless, the capacity to generate porous scaffolds.

hADSCs inside these novel IPNs were able to populate the entire scaffold structure and to display their characteristic spindle-*like* shape as a consequence of a good interaction with G component of the matrices. Additionally, hADSCs proved to display the capacity to differentiate towards mature adipocytes, to accumulate lipids inside their cytoplasm, and to express perilipin late adipogenic marker inside novel IPNs described in this study. Consequently, the presence of the synthetic PAA component in the formulation of the scaffold did not influence either hADSCs behavior in contact with the material or the evolution of the adipogenic differentiation process, but it ensured a more appropriate 3D microenvironment for cells, resembling* in vivo* conditions. Furthermore, as a perspective for tissue engineering advance, hADSCs/G-A-PAA could be an eligible candidate as cellular delivery system at the injury sites for regenerative purposes.

## Figures and Tables

**Table 1:** Primer sequences used for adipogenic marker identification by qPCR.

Target	Nucleotide sequence
Perilipin F	5′-ATGCTTCCAGAAGACCTACA-3′
Perilipin R	5′-CAGCTCAGAAGCAATCTTTT-3′
TBP F	5′-AGGCATCTGTCTTTGCACAC-3′
TBP R	5′-GGGTCAGTCCAGTGCCATAA-3′
RPL13A F	5′-CCTGGAGGAGAAGAGGAAAGAGA-3′
RPL13A R	5′-TTGAGGACCTCTGTGTATTTGTCAA-3′

TBP: TATAA-box binding protein; RPL13A: ribosomal protein L13A.
